# The geometric preference subtype in ASD: identifying a consistent, early-emerging phenomenon through eye tracking

**DOI:** 10.1186/s13229-018-0202-z

**Published:** 2018-03-21

**Authors:** Adrienne Moore, Madeline Wozniak, Andrew Yousef, Cindy Carter Barnes, Debra Cha, Eric Courchesne, Karen Pierce

**Affiliations:** Autism Center of Excellence, Department of Neurosciences, University of California San Diego, La Jolla, CA USA

**Keywords:** Eye tracking, Autism spectrum disorder, Early identification, Social attention, Geometric preference

## Abstract

**Background:**

The wide range of ability and disability in ASD creates a need for tools that parse the phenotypic heterogeneity into meaningful subtypes. Using eye tracking, our past studies revealed that when presented with social and geometric images, a subset of ASD toddlers preferred viewing geometric images, and these toddlers also had greater symptom severity than ASD toddlers with greater social attention. This study tests whether this “GeoPref test” effect would generalize across different social stimuli.

**Methods:**

Two hundred and twenty-seven toddlers (76 ASD) watched a 90-s video, the Complex Social GeoPref test, of dynamic geometric images paired with social images of children interacting and moving. Proportion of visual fixation time and number of saccades per second to both images were calculated. To allow for cross-paradigm comparisons, a subset of 126 toddlers also participated in the original GeoPref test. Measures of cognitive and social functioning (MSEL, ADOS, VABS) were collected and related to eye tracking data. To examine utility as a diagnostic indicator to detect ASD toddlers, validation statistics (e.g., sensitivity, specificity, ROC, AUC) were calculated for the Complex Social GeoPref test alone and when combined with the original GeoPref test.

**Results:**

ASD toddlers spent a significantly greater amount of time viewing geometric images than any other diagnostic group. Fixation patterns from ASD toddlers who participated in both tests revealed a significant correlation, supporting the idea that these tests identify a phenotypically meaningful ASD subgroup. Combined use of both original and Complex Social GeoPref tests identified a subgroup of about 1 in 3 ASD toddlers from the “GeoPref” subtype (sensitivity 35%, specificity 94%, AUC 0.75.) Replicating our previous studies, more time looking at geometric images was associated with significantly greater ADOS symptom severity.

**Conclusions:**

Regardless of the complexity of the social images used (low in the original GeoPref test vs high in the new Complex Social GeoPref test), eye tracking of toddlers can accurately identify a specific ASD “GeoPref” subtype with elevated symptom severity. The GeoPref tests are predictive of ASD at the individual subject level and thus potentially useful for various clinical applications (e.g., early identification, prognosis, or development of subtype-specific treatments).

**Electronic supplementary material:**

The online version of this article (10.1186/s13229-018-0202-z) contains supplementary material, which is available to authorized users.

## Background

Autism spectrum disorder (ASD) encompasses a heterogeneous collection of phenotypes. Some individuals with ASD are highly capable, verbally fluent individuals who view their autism as a benign difference requiring an increase in tolerance and acceptance from the neurotypical community rather than a cure [1]. Others with ASD are severely impaired with minimal ability for self-care or for communicating their perspectives or needs [2–4]. It may be possible to maximize impact of treatment for those with particularly challenging forms of ASD by intervening early in the development of symptoms [5–7]. Neurobiological differences between people who will go on to be diagnosed with ASD and those who will not have been traced back to even prenatal stages of development [8–10]. Differences in behavioral presentation at the group level between children who will and will not go on to be diagnosed with ASD have been found as early as 6 months [11–13]. However, according to recent Centers for Disease Control and Prevention reporting, most children on the autism spectrum in the USA are not diagnosed until after the age of 4 [14]. The development of effective tests that can reliably identify in their infancy which individuals will go on to be diagnosed with autism, and whether that autism will ultimately be mild or severe, is in its very early stages.

Clinician judgments of observed behavior, though vulnerable to subjective bias, remain the gold standard for ASD identification [15]. This state of affairs persists despite widespread acceptance that the origins of ASD are neurobiological and that therefore the development of highly objective tests should be achievable [16]. Eye tracking is a methodology with great potential clinical utility for screening, diagnosis, and early detection of ASD [17]. It is objective, quantitative, non-invasive, relatively inexpensive and easy to use, and appropriate for very young infants and many levels of functioning [18, 19]. Moreover, while basic oculomotor functioning has been shown not to differ in fundamental ways [19] between ASD and controls (although see [20, 21] for notable differences in spontaneous fixation durations and attentional disengagement), patterns of viewing socially relevant information reveal the phenotypic differences between ASD and typical development [22]. When paired with stimuli and tasks that have been well explored by the field of neuroscience, eye tracking may move us toward clinical approaches grounded in knowledge of the disrupted neural circuitry of ASD, with the goal of improved treatment impact [23].

Eye tracking studies of toddlers and young children with ASD have reported less time attending to biological motion [24], less attention to people’s heads and more to bodies [25], less time viewing people and faces within a complex scene [13], and, when viewing faces, less time spent viewing the key feature components [26]. Young children with ASD also exhibit atypical gaze-following behavior during eye tracking paradigms [27] which is important because gaze-following is a key precursor to the development of joint attention [28]. Joint attention skills are critically associated with language acquisition in typically developing children [29] and with language and social deficits in ASD [30]. Though it has not been demonstrated, the social differences and difficulties of adults with ASD could possibly be influenced by the long-term, cumulative impact of this abnormal visual attention to what is socially meaningful during development [31–33]. Abnormal non-social attentional components (e.g., disengagement) likely add complexity to this explanation as well [34]. Intervention studies focused on improving joint attention skills have yielded promising results thus far, suggesting it may be feasible to alter this course of events as ASD unfolds across childhood [35].

Despite the many insights into ASD development stemming from eye tracking research, difficultly when comparing results from different eye tracking studies of ASD toddlers has been noted [22]. This is in part because seemingly minor changes to the stimuli presented may alter the results considerably. For example, in separate studies, Jones [31] and Chawarska [36] presented video stimuli to ASD toddlers of similar ages (mean age 2.1 years, standard deviation .65 years, and the 13- to 25-month age range, respectively). Both studies included a complex stimulus background with toys and other objects, in front of which was a centrally located female actress who looked directly into the camera while trying to attract attention with child-directed speech. However, Jones (2008) found increased looking to the mouth region and decreased looking to the eye region in ASD children compared to contrast groups. Chawarska, on the other hand, found decreased looking time to the face and specifically to the mouth region and increased looking to the hands in ASD children, yet no differences in eye region fixation per se, compared to contrast groups. There are various possible explanations for this discrepancy, and several authors have previously commented on it [22, 37]. Regardless of the cause, the sharp inconsistency of findings between such studies suggests that results when eye tracking toddlers with ASD can be very sensitive and may not generalize robustly or replicate unless many factors are controlled.

**Fig. 1:**
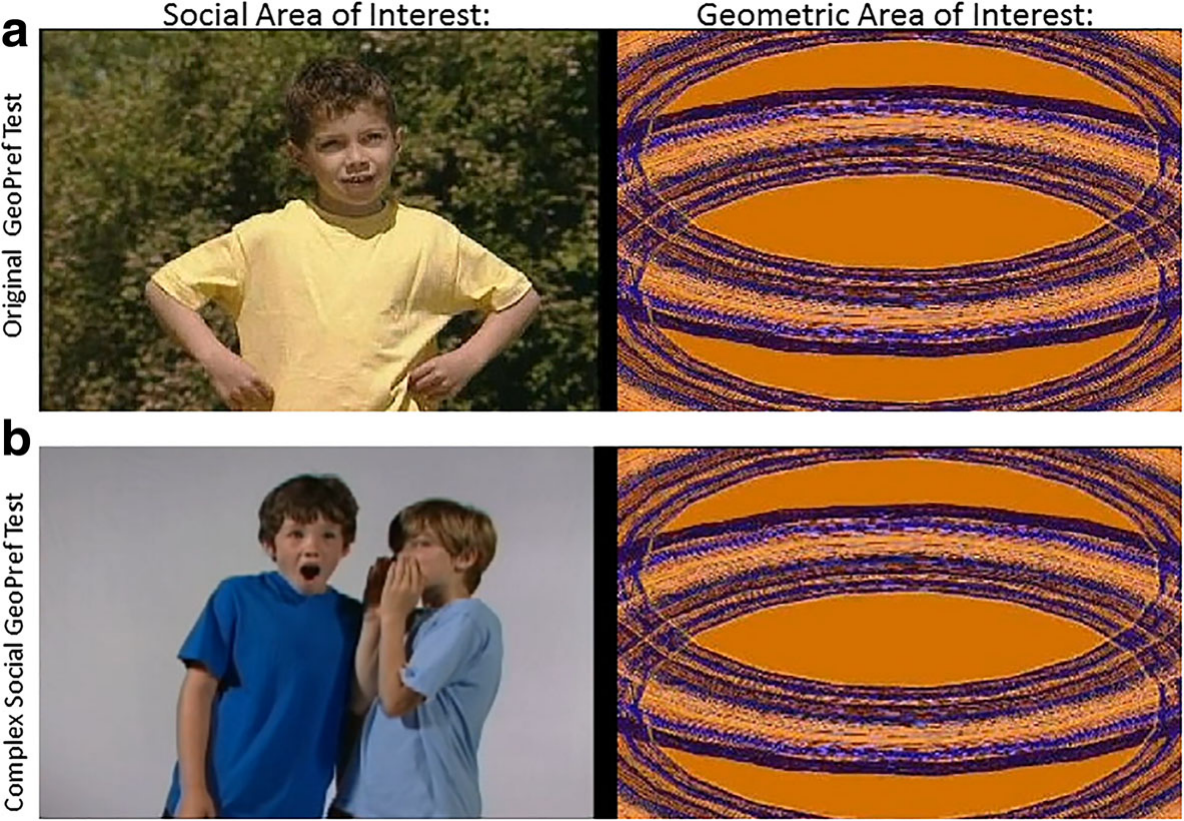
Examples of stimuli. **a** Sample image from the original GeoPref test which consists of a 60-s video composed of 28 scenes, 26 of which involve one individual’s movement (while the other two scenes include twins moving side by side). ©2003 Gaiam Americas, Inc., Courtesy of Gaiam Americas, Inc. As each social scene switches to a new actor, the paired geometric scene switches simultaneously to different colors and moving shapes. **b** Sample image from the Complex Social GeoPref test which consists of a 90-s video composed of nine scenes, five of which include two children interacting and four of which include one child moving enthusiastically. As each social scene changes, the paired geometric scene also switches simultaneously to a different color and moving shape, as in the original GeoPref test

In contrast, the current eye tracking study examines whether the GeoPref test effect is robust against changes to the social images presented in a conceptual replication of the “GeoPref” subtype effect identified in our previous work (see Fig. 1). Specifically, in 2011, Pierce et al. reported eye tracking data from a preferential looking task showing that preference for viewing geometric rather than social stimuli is a risk factor for an autism diagnosis in toddlers [38]. In 2016, Pierce et al. reported that this Geometric Preference (GeoPref) test identifies an ASD subtype with increased symptom severity compared to ASD children who preferentially view social images [39]. Individuals in the GeoPref ASD subtype, who spent more than 69% of looking time viewing geometric stimuli, had higher ADOS scores, lower MSEL receptive and expressive language and visual reception scores, and lower Vineland scores for adaptive behavior.

The current study tests the Geometric Preference phenomenon identified previously by varying the social stimulus presentation’s total length, scene length, and complexity of social interactions. The original study shows full body or large, dance-like movements and uniformly positive affect. The current study depicts a broader range of expressed emotions including surprise and anger, as well as happiness, and shows socially meaningful but physically more subtle actions like whispering in another’s ear, hugging, and one child sticking out her tongue at another. As in the original test, these social stimuli portray biological motion and faces (though with less biological motion and more varied facial expressions), keeping the paradigm closely linked to stimuli that have been often used by cognitive neuroscience in attempts to map the social brain [40–42]. These complex social vignettes unfold more gradually than the actions in the original stimuli; therefore, the stimulus video is longer overall and composed of longer individual scenes. The geometric stimuli were not altered, other than by selecting a subset and extending the duration of presentation per scene to match the durations to the social stimuli. This was done so that by isolating the social variables only, we could conclude that changes in the pattern of responses to viewing the stimuli were due to the social scene manipulation, thus avoiding any confounds. Because toddlers with ASD are more likely to have a reduced interest in social stimuli, we considered the fact that the sensitivity of the test (which was around 23% for the original GeoPref test) might improve if we altered the complexity of the social stimuli. That is, we predicted that a greater percentage of ASD toddlers may find the social side uninteresting (and would thus fixate on the geometric images instead) if it were made more complex. However, for typical toddlers, it may increase their interest in the social side if social interactions were depicted, which would potentially increase group differences.

In a meta-analysis of 38 articles comparing ASD and TD children using eye tracking [43], Chita-Tegmark reports that increasing the social content of stimuli by showing more than one person is the factor that best reveals the differences in social attention between ASD and TD groups. We tested the hypothesis that the original GeoPref test identifies a stable subtype of autism characterized by robust patterns of decreased social attention and increased attention to geometric repetition and therefore that the Complex Social GeoPref test should generally replicate the findings of the original GeoPref test, perhaps with amplified effects due to changes to the social stimuli used. That is, ASD children were predicted on average to have greater fixation times on geometric images than contrast groups, and above some threshold, all children with sufficiently high fixation time on geometric images were predicted to be ASD children. Further, ASD children who complete both tests within the appropriate age range were predicted to have fairly stable scores. Additionally, the ASD children with greatest fixation times on geometric images, the GeoPref ASD subtype, were predicted to also have worse cognitive, language, and social skills based on MSEL, ADOS, and VABS scores compared with ASD children with the least geometric fixation times (the ASD “SocPref” subtype).

## Methods

### Participant recruitment

Two hundred and seventy toddlers enrolled in and completed this study. Of the 270, 43 were excluded from data analysis for reasons detailed in Additional file 1: Figure S1 (e.g., vision abnormality, tantrum during eye tracking), leaving a final study sample of 227 toddlers. Their ages ranged from 12 to 48 months (mean 29.5 months, standard deviation 9.5). Two hundred and eleven of the 227 subjects in the present study (93%) were new and non-overlapping with our past two eye tracking papers [38, 39]. Of the 227 participants, 126 completed both the Complex Social GeoPref eye tracking test newly described herein and the original GeoPref test described in previous publications [38, 39, 44]. Sixty-eight of these 126 (54%) completed the original GeoPref test first, and 58 (46%) completed the Complex Social GeoPref test first, and no age differences were found between groups at either time point. The remaining 101 subjects completed the new Complex Social GeoPref test but not the original GeoPref test.

All diagnostic, psychometric and eye tracking tests took place at the University of California San Diego Autism Center. During data collection time periods, any child receiving an autism evaluation, regardless of referral source, was included in eye tracking testing. Fifty-four percent of the sample of 227 were referred to us by their pediatrician who participates in our general population-based screening method called the 1-Year Well-Baby Check-Up Approach [44]. This allows for the prospective study of ASD, as well as global developmental and language delay or other delays, beginning as early as 12 months, typically based on a toddler’s failure of the CSBS-DP Infant-Toddler Checklist [45]. Occasionally, a child is referred by a participating pediatrician between ages 2 and 3 so the CSBS is no longer applicable, or because there is concern regarding the child’s development despite a passing score on the CSBS questionnaire. The remaining 46% of subjects were not referred by their pediatricians. These participants either self-referred due to parental concern about their child’s development, or participated as controls. Though they were not referred after pediatrician screening for developmental delays, these children received identical testing to the screening referred group during their evaluations at the UCSD Autism Center. ASD children comprise 38% of the group referred through pediatrician screening and 29% of the self-referred group, and this difference falls short of statistical significance (chi-squared = 2.09, *p* = .15). Mean age at eye tracking for the pediatrician screening referred group was 29.9 months; mean age for the self-referred group was 29.0 months at eye tracking.

### Diagnostic and psychometric assessments

At each visit, assessments were administered at UCSD Autism Center by PhD-level licensed clinical psychologists and included the Autism Diagnostic Observation Schedule (ADOS) module T, 1, or 2 [46], Mullen Scales of Early Learning (MSEL) [47], and Vineland Adaptive Behavior Scales (VABS) [48]; additional family and medical histories were also obtained. Toddlers who participated when younger than age 30 months were longitudinally tracked and diagnostically evaluated every 6–12 months until age 3 years when a final diagnosis was given. Any child receiving an evaluation during the data collection time period was administered eye tracking for this study, regardless of whether their visit was an intake appointment, a follow-up, or a final diagnostic appointment. Table 1 presents characteristics of the sample.

**Table 1:** Participant characteristics of overall sample

	1) ASD^a^	2) ASD feat.	3) DD	4) TD	5) Other	6) Typical sibling ASD	ASD vs 2), *p*=	ASD vs 3), *p*=	ASD vs 4), *p*=	ASD vs 5), *p*=	ASD vs 6), *p*=
Sex, M/F	70/6	10/1	36/20	30/21	11/11	4/7	n/a	n/a	n/a	n/a	n/a
Age at eye tracking, months Mean (SD) [range]	30.0 (8.8) [12.1–47.4]	31.9 (8.9) [15.8–40.7]	26.8 (9.5) [12.4–46.0]	29.7 (9.5) [12.9–47.5]	33.6 (10.3) [13.1–47.7]	27.8 (11.2) [12.2–44.6]	NS	NS	NS	NS	NS
MSEL AE^b^ scores/true age											
Visual reception	.79 (.18)	.94 (.24)	.93 (.19)	1.16 (.18)	1.05 (.20)	1.17 (.16)	NS	< .001	< .001	< .001	< .001
Fine motor	.79 (.18)	.90 (.13)	.96 (.18)	1.04 (.13)	.95 (.20)	1.04 (.16)	NS	< .001	< .001	< .01	< .001
Receptive language	.58 (.28)	.82 (.24)	.79 (.24)	1.10 (.16)	1.0 (.23)	1.03 (.15)	< .05	< .001	< .001	< .001	< .001
Expressive language	.60 (.26)	.72 (.20)	.64 (.23)	1.04 (.17)	.99 (.25)	1.04 (.13)	NS	NS	< .001	< .001	< .001
VABS^c^ standard scores											
Communication	75.2 (18.8)	90.8 (17.2)	84.6 (15.4)	104.1 (9.8)	98.8 (12.0)	101.5 (5.1)	< .05	< .05	< .001	< .001	< .001
Daily living	82.4 (15.5)	99.2 (11.7)	92.2 (13.2)	101.1 (9.3)	96.7 (15.2)	98.4 (11.6)	< .005	= .001	< .001	< .001	< .005
Socialization	80.1 (16.1)	96.0 (11.3)	96.4 (11.2)	102.7 (9.2)	97.4 (13.7)	104.4 (6.6)	< .005	< .001	< .001	< .001	< .001
Motor skills	87.8 (17.3)	96.6 (10.4)	91.0 (13.4)	99.7 (9.6)	94.8 (13.3)	99.9 (6.5)	NS	NS	< .001	NS	NS
Composite score	78.8 (15.8)	94.6 (11.7)	88.6 (11.2)	102.7 (10.4)	96.1 (13.1)	100.9 (5.7)	< .005	< .001	< .001	< .001	< .001
ADOS^d^ module T, 1, or 2											
SA^e^	13.2 (4.2)	6.2 (5.5)	4.3 (3.1)	2.5 (1.7)	4.3 (3.7)	3.1 (2.2)	< .001	< .001	< .001	< .001	< .001
RRB^f^	3.7 (2.0)	2.0 (1.5)	0.8 (1.8)	0.4 (0.8)	0.4 (0.6)	0.5 (0.5)	< .05	< .001	< .001	< .001	< .001
Total score	16.9 (5.2)	8.2 (6.0)	5.2 (3.8)	2.9 (2.0)	4.7 (4.0)	3.6 (2.0)	< .001	< .001	< .001	< .001	< .001

^a^See text for descriptions of diagnostic groups ASD, ASD Feat., DD, TD, Other, Typical Sibling ASD

^b^Mullen Scales of Early Learning, Age Equivalent

^c^Vineland Adaptive Behavior Scales

^d^Autism Diagnostic Observation Schedule

^e^Social Affect Score

^f^Restricted and Repetitive Behaviors Score

The study sample consisted of six discrete diagnostic groups of toddlers: 76 ASD, 11 ASD features, 56 DD, 51 TD, 22 Other, and 11 TypSib. The *ASD* group included toddlers who met DSM criteria for Autistic Disorder or PDD-NOS (DSM IV) or ASD (DSM V) at their final diagnostic evaluation. The *ASD features* group had significant ASD symptoms and/or elevated ADOS scores during at least one evaluation but did not meet full criteria for ASD at their final longitudinal evaluation. The *DD* group included transient and persistent language delay and global developmental delay determined by MSEL scores. The *TD* group included “type 1 errors,” children who failed the CSBS screening at a pediatric visit but tested within typical levels on ADOS, MSEL, and VABS during their evaluations, as well as typically developing toddlers who both passed the CSBS and tested within the typical range on ADOS, MSEL, and VABS tests during their evaluations. In the *TypSib* group were unaffected toddlers with siblings with ASD who tested within the typical range during their evaluations. In the *Other* group were toddlers with a wide array of other conditions such as social anxiety or a tic disorder. For this study, 83% of the overall sample received a final diagnostic assessment at 30 months or older (mean age 38.3 months). The remaining 17% (*13 ASD*, *18 DD*, *4 TD*, *2 TypSib*, *1 Other*) were assigned to a diagnostic group based on a diagnosis given between 18 and 30 months (mean age 24.2 months).

### Movie, apparatus, and eye tracking procedure

The Complex Social GeoPref test contained a 90-s movie composed of two large, rectangular areas of interest (AOIs) side by side (see Fig. 1) where one AOI displayed geometric patterns and the other social scenes. There was no audio. The geometric patterns were a subset of those of the original 60-s GeoPref test [38]; however, each geometric pattern was repeated for a longer time interval to achieve a 90-s test. Each social scene was paired with one of the moving, colorful geometric patterns, and when each social scene changed, each geometric pattern also changed. The social scenes included five scenes showing two children interacting. The interactions were dance-like twisting side by side, jumping to a high-five, whispering a secret then appearing surprised, whispering a secret then hugging, and teasing by sticking out tongues then stomping on the other’s foot. To allow for cross-paradigm comparisons, a subset of 126 toddlers participated in the original GeoPref test identical to Pierce et al. [38] as well as the new Complex Social GeoPref test on separate visits. Unlike the complex social scenes in the present design, in the original GeoPref test, all scenes similarly showed children doing rhythmic, dance-like movements all displaying a uniformly positive affect. See Fig. 1 for sample images and the movie clip “Additional file 2” for more details.


Additional file 2:Stimulus Video Example. (MP4 14305 kb)


Eye tracking data were collected while toddlers, seated on a parent’s lap, viewed these videos from 60 cm distance on a 17″ thin-film transistor monitor using the Tobii T-120 system set at 60 Hz. Five-point calibration was first performed with Tobii Studio software using an animated image with sound presented at known *X*-*Y* coordinates. Eye tracking data were collected only if the calibration result fell within the parameters reported by the manufacturer to yield an accuracy of 0.5° [49]. Each AOI subtended 12.9° horizontally and 9.1° vertically. This use of large, simple AOIs facilitated correct measurement of the infant/toddler population, who can yield data with accuracy below levels reported for adults under optimal conditions [50]. More information about data spatial accuracy is provided in the Additional file 1.

Fixations were classified based on gaze data averaged from both eyes using a velocity threshold Tobii Fixation Filter set to 35 pixels/window, which interpolates to fill in data loss of less than 100 ms. For each subject, number of fixations, duration of each fixation, and sum of fixation time within the two AOIs (social and geometric) were calculated. Sum of fixation time per AOI was divided by total sum of fixation time for both AOIs to derive proportion of time spent on each AOI (i.e., “%Geo” and “%Soc”) and to correct for missing data. Subjects with excessive missing data (i.e., less than 30 s of data) due to attending to neither AOI or due to inability to track eye gaze (e.g., during excessive movement) were excluded, in order to preclude inaccurate measurement of number or length of fixations and saccades. Number of fixations per AOI was divided by sum of fixation time for that AOI to derive saccade frequency as saccades per second, which was also reported in our prior publication [39]. See the Additional file 1: Figure S1 for the complete description of exclusion criteria and lab practices for assuring data accuracy and precision, and also the results regarding saccade per second differences between groups.

### Statistical analyses

#### Percent of total fixation duration to geometric vs complex social stimuli

No age differences were found between diagnostic groups (one-way ANOVA with no overall effect of age; see Table 1). To compare percentage of total fixation time within the geometric AOI between groups, a one-way ANOVA was performed (diagnostic group (6 levels) × %Geo (1 level)). A significant main effect was followed by Bonferroni-corrected post hoc pairwise comparisons. Prior to selecting these analysis strategies, homogeneity of variance was confirmed with Levene’s test. To confirm that differences in data quality between diagnostic groups were not impacting the reported results, an ANCOVA was performed as well, with six diagnostic groups as a fixed factor, %Geo as the dependent variable, and a data quality measure (percent of valid samples obtained) as a covariate. There was no significant effect of the data quality metric (*F*_5,221_ = .011, *p* = .916).

#### Relationships between percent of total fixation duration and clinical characteristics

All statistical values for clinical scores presented in Table 1, namely, those of ADOS, MSEL, and VABS, were calculated in the same manner: one-way ANOVAs were performed with post hoc pairwise tests and Bonferroni corrections. The relationship between Complex Social GeoPref %Geo scores and ADOS total scores was assessed with Spearman’s rank-order correlations. After identifying the ASD children with the strongest preferences for geometric and for social images using %Geo and %Soc scores, within ASD analyses focused on differences in clinical characteristics between the subtypes “GeoPref” and “SocPref” followed strategies like those described above: homogeneity of variance was assessed and no significant age difference between groups was found, so independent samples *t* tests were used to compare scores on ADOS, MSEL, and VABS scores. These comparisons presented in Table 2 are reported one-tailed based on a priori hypotheses from our 2016 manuscript regarding the direction of differences. Correction for multiple comparisons was performed using the Benjamini-Hochberg procedure. Cohen’s *d* effect sizes are reported as well.

**Table 2:** Participant characteristics of ASD subgroups

	ASD GeoPref subtype	ASD SocPref subtype	*t* test, corrected *p*	Cohen’s *d*
Sex, M/F	13/1	15/2	n/a	
Age at eye tracking, months Mean (SD) [range]	31.2 (9.2) [13.8–47.2]	28.7 (7.2) [16.4–45.4]	.40	.30
MSEL^a^ age equivalent scores/true age				
Visual reception	.76 (.17)	.81 (.23)	.50	.25
Fine motor	.76 (.19)	.82 (.18)	.41	.32
Receptive language	.46 (.25)	.68 (.26)	.03	.86
Expressive language	.53 (.26)	.67 (.30)	.19	.50
VABS^b^ standard scores				
Communication	71.0 (16.0)	81.5 (16.0)	.08	.66
Daily living	80.9 (15.2)	84.1 (15.3)	.57	.21
Socialization	77.1 (12.7)	84.8 (14.4)	.13	.57
Motor skills	84.4 (10.7)	95.7 (15.0)	.03	.87
Composite score	75.6 (12.7)	84.2 (15.9)	.11	.60
ADOS^c^ module T, 1, or 2				
SA^d^	16.7 (3.2)	11.4 (4.1)	< .001	1.4
RRB^e^	4.2 (2.5)	3.1 (1.7)	.15	.51
Total score	20.9 (4.4)	14.5 (4.2)	< .001	1.5

^a^Mullen Scales of Early Learning

^b^Vineland Adaptive Behavior Scales

^c^Autism Diagnostic Observation Schedule

^d^Social affect

^e^Restricted and repetitive behaviors

#### Clinical classification performance: sensitivity, specificity, PPV, NPV, and ROC curve

To assess the ability of the Complex Social GeoPref test to discriminate toddlers with ASD from other toddlers, sensitivity, specificity, positive predictive value (PPV), negative predictive value (NPV), and Receiver Operating Characteristic (ROC) area under the curve were determined. For consistency and comparison with our past publications, 69% looking time to geometric images was used as the cut-off for a positive result. Although PPV and NPV for a general population ASD screening tool would be calculated based on the 1/68 prevalence rate for ASD [14], the GeoPref tests are best suited as second tier tools, administered after a questionnaire screener which has higher sensitivity but lower specificity. Therefore, PPV and NPV were calculated here against the ASD rate in our sample (i.e., 1/3). This rate reflects a PPV and NPV that might be expected at a general ASD and developmental disorder diagnosis and evaluation clinic, where children are referred primarily due to failing a first-tier screening tool (i.e., the CSBS-DP Infant Toddler Checklist). Classification statistics are presented separately for the entire sample and for screening referred children only without including self-referred children. However, it is to be expected that in a real-world clinical setting, self-referrals will naturally occur as there are many ways outside of pediatrician screening (e.g., Google searching) that community members might become aware of the availability of evaluation services and then self-refer.

Because the greatest challenge to clinicians is distinguishing ASD toddlers from toddlers with other sorts of delays, these classification performance measures were also calculated without the inclusion of TD and TypSib groups and are presented in the Additional file 1. Because 69% was chosen in our previous work by setting the test’s specificity to 99%, classification performance values are also reported for the cut-off that gives a specificity of 99% on the Complex Social GeoPref test (75% of looking time to geometric images) in the Additional file 1. Use of the Complex Social GeoPref test in order to rule out a diagnosis of ASD, where having a %Geo score below a certain threshold is considered positive, plus having any diagnosis other than ASD is considered true positive, is examined in the Additional file 1 with regard to sensitivity, specificity, PPV, NPV, and AUC.

#### Comparing and combining of complex social and original GeoPref tests

Differences between the Complex Social GeoPref and original GeoPref tests for the subset of children who completed both tests in the percentage of time viewing geometric stimuli (%Geo scores) were investigated in several ways. Paired samples *t* tests were used to compare %Geo scores for the two tests for each diagnostic group. Degree of correlation between test scores for individual children who completed both tests was assessed with Spearman’s rank-order correlation. Use of both tests by a single child, where a positive score on either test (or both tests) is considered a positive result, was also examined with regard to sensitivity, specificity, PPV, NPV, and AUC. AUC for this two-test model was determined based on predicted probabilities calculated using binary logistic regression with %Geo scores for the two tests as covariates.

## Results

### Percent of total fixation duration of the six diagnostic groups to geometric vs complex social stimuli

**Fig. 2:**
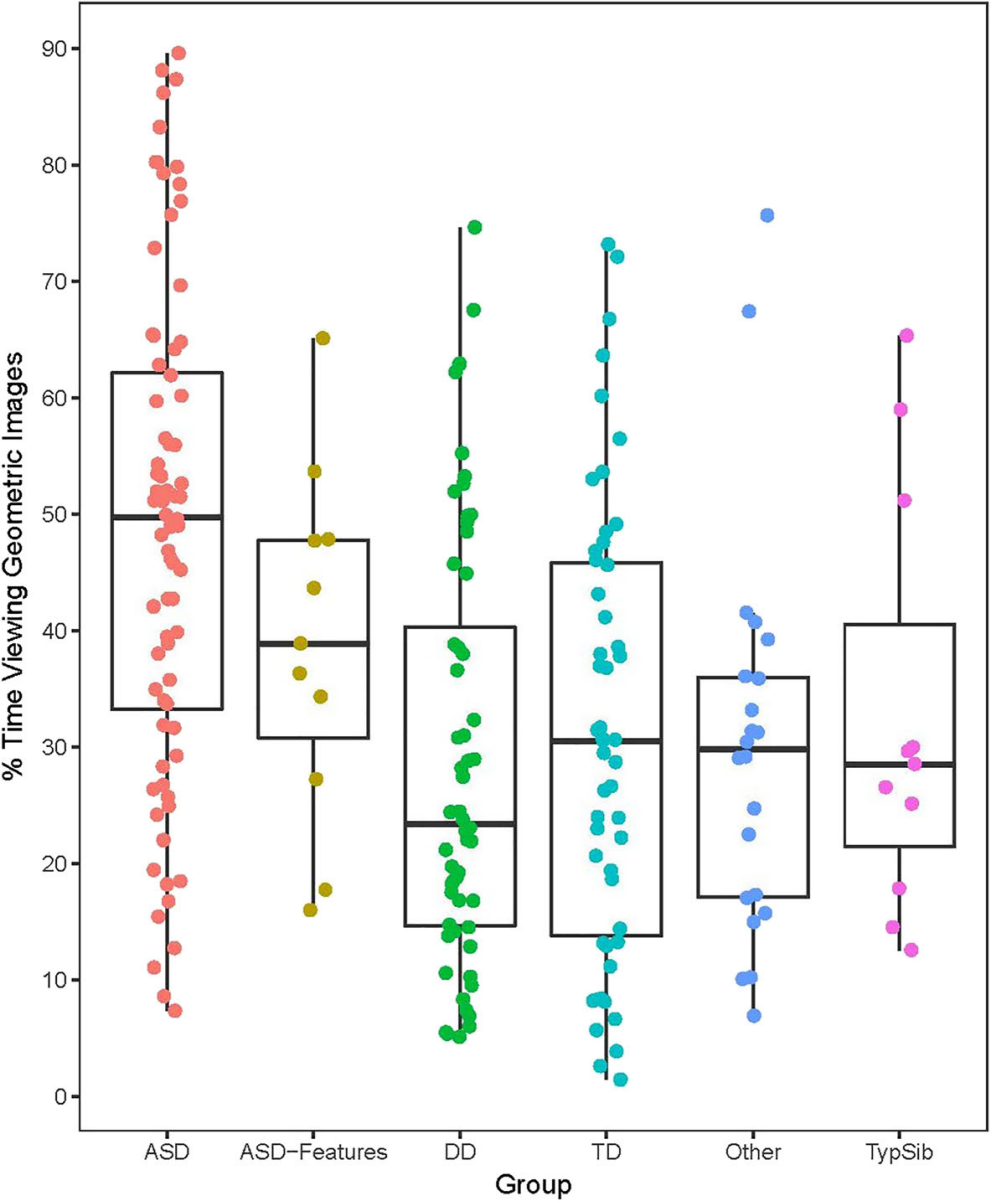
Scatterplot of % time viewing geometric images (total fixation duration while viewing the dynamic geometric stimulus, divided by total fixation duration to the geometric and social stimuli combined) for all subjects who completed the Complex Social GeoPref test (*n* = 227) sorted by diagnostic group. Boxplots show median, range, and first and third quartiles

In our new Complex Social GeoPref test, geometric images attracted significantly more looking time in ASD than in TD, DD, and other groups (*F*_5,221_ = 9.1, *p* < .001, partial eta-squared = .17; ASD vs TD, *p* < .001, Cohen’s *d* = .85; ASD vs DD, *p* < .001, Cohen’s *d* = 1.0; ASD vs other, *p* < .005, Cohen’s *d* = .96). Toddlers with ASD spent an average of 48.4% of their time looking at geometric images (95% confidence interval (CI) range 43.6–53.2%); TD toddlers 31.2% of their time (95% CI 25.8–36.6%); DD toddlers 28.6% of their time (95% CI 23.8–33.4%); and other toddlers 30.0% of their time (95% CI 22.5–37.6%). Toddlers with ASD also looked more at geometric images than TypSibs (mean 32.8%, 95% CI 20.8–44.7%) and ASD features (mean 39.0%, 95% CI 28.9–49.0%), but these differences were not statistically significant, perhaps due to small sample sizes in the latter two study groups. See Fig. 2.

### Within ASD: differences between the ASD GeoPref and ASD SocPref subtypes

#### Percent of total fixation duration per AOI and clinical characteristic comparisons

In order to compare clinical characteristics associated with ASD toddlers at either end of the fixation spectrum [39] (i.e., those who strongly preferred geometric images and those who strongly preferred social images), ASD toddlers were identified who were either the ASD GeoPref subtype (> 69% time looking at geometric images) or the ASD SocPref subtype (> 69% time looking at complex social images). The mean %Geo looking score for the ASD GeoPref subtype was 80.5% (or 19.5% social looking), and the mean %Soc looking score for the ASD SocPref subtype was 80.3% (or 19.7% geometric looking).

Clinical differences between the two ASD subtypes are shown in Table 2. Similar to our previous reports [39], as compared to the ASD SocPref subjects, the ASD GeoPref subjects had significantly increased ADOS social affect (*t*_29_ = 4.0, *p* < .001, Cohen’s *d* = 1.4) and total scores (*t*_29_ = 4.2, *p* < .001, Cohen’s *d* = 1.5). Further, across the entire group of 76 toddlers with ASD, %Geo scores on the Complex Social GeoPref test were significantly correlated with ADOS total scores, that is, those with greater preference for looking at the geometric stimuli had more severe autism scores (Spearman’s rho = .43, *p* < .001), and this is presented in the Additional file 1. The ASD GeoPref subjects appeared to also have lower mean Mullen receptive language scores, and lower Vineland motor scores, with moderate Cohen’s *d* effect sizes.

### New Complex Social GeoPref test vs original GeoPref test

Eye tracking data were examined from 126 of the 227 study subjects (37 ASD, 10 ASD features, 30 DD, 32 TD, 7 Typ-Sibs, 10 other) who completed both the new Complex Social GeoPref test and the original GeoPref test on separate visits to the center. Sixty-seven completed the original GeoPref test first (mean age 27.3 months at original GeoPref testing) and 59 completed the Complex Social GeoPref test first (mean age 29.0 months at Complex Social GeoPref testing), and this age difference was not significant. Further, no diagnostic group differences in age at testing were found.

Across all diagnostic groups, the mean difference between the Complex Social GeoPref and original GeoPref tests in percent time looking at geometric images (%Geo) was 1.3% (%Geo = 35.4% for the Complex Social vs %Geo = 34.1% for the original GeoPref test), and this was not significant (*t*_125_ = 0.6, *p* = .60). For the ASD group, the mean %Geo score was 48.7% for the Complex Social and 44.7% for the original GeoPref tests, which was a non-significant difference (*t*_35_ = 1.0, *p* = .31).

**Fig. 3:**
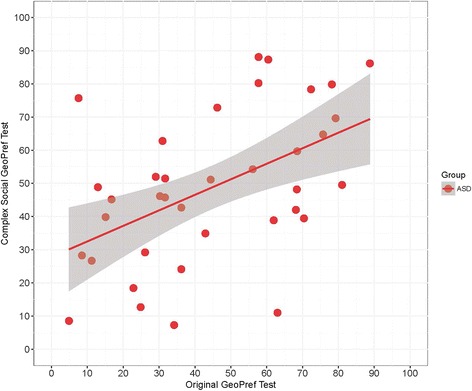
Correlation between original and Complex Social GeoPref Tests. Scatterplot illustrating % Geo scores (summed fixation duration while viewing the dynamic geometric stimulus, divided by total fixation duration to the geometric and social stimuli combined) for each test for each subject in the ASD group who completed both eye tracking tests on separate visits across the span of the study

There was a significant within-subject correlation in %Geo of total fixation duration across the Complex Social and original GeoPref tests across all study subjects (*N* = 126; Spearman’s *r* = .25, *p* < .005) and within the ASD group (*N* = 37; Spearman’s *r* = .47, *p* < .005). See Fig. 3. For TD and DD groups, these scores were not significantly correlated.

ROC comparisons were all examined, with rates of specificity, sensitivity, PPV, NPV, and AUC compared between the Complex Social and original GeoPref tests in Table 3. AUC was 0.74 for the Complex Social GeoPref test. For comparison, AUC was 0.71 for the original GeoPref test in Pierce et al. (2016). Removal of typically developing children from analysis and focusing on children with some sort of delay or disorder further improved AUC to 0.75 (see Additional file 1). Removal of self-referred children and focusing on screening referred only yielded an AUC of 0.73.

**Table 3:** Clinical classification performance, comparison of original and Complex Social Geopref tests

69% Geo threshold	All available subjects *N* = 444	All available subjects *N* = 227	Screening referred subjects only *N* = 122
True positive = ASD only	Original GeoPref test (2016)	Complex Social GeoPref test	Complex Social GeoPref test
True positive	35	14	10
False negative	117	62	36
False positive	4	4	3
True negative	288	147	73
Sensitivity (%)	23	18	22
Specificity (%)	99	97	96
Positive predictive value (%)	90	78	77
Negative predictivevalue (%)	71	70	67
Area under ROC curve	.71	.74	.73

### Clinical classification performance: combination of new and original GeoPref tests

AUC, sensitivity, specificity, and PPV and NPV values were then examined when using a two-test screening model on data from the 126 subjects who participated in both the Complex Social and original GeoPref tests in order to determine if use of two tests enhanced classification performance. Results are shown in Table 4 (see data in the left two columns). Sensitivity increased substantially from 18% for the Complex Social test alone (Table 3) to 35% with two tests, while specificity remained high. AUC calculated for this two-test model was 0.75. Pilot results in the Additional file 1 suggest this enhancement does not occur if the two tests are given immediately back-to-back but only if separated in time.

**Table 4:** Clinical classification, two tests: combined original and Complex Social Geopref Tests

69% Geo threshold	All available subjects *N* = 126	All available subjects *N* = 126	Screening referred subjects only *N* = 82	Screening referred subjects only *N* = 82
Positive = positive on either test	ASD only = true positive	ASD + ASD features = true positive	ASD only = true positive	ASD + ASD features = true positive
True positive	13	15	9	11
False negative	24	32	18	23
False positive	5	3	5	3
True negative	84	76	50	45
Sensitivity (%)	35	32	33	32
Specificity (%)	94	96	91	94
Positive predictive value (%)	72	83	64	79
Negative predictive value (%)	78	70	74	66
Area under ROC curve	.75	.78	.73	.76

Classification performance of the combination of the two tests for screening referred toddlers only was also examined. Results are shown in Table 4 (data in the right two columns). Again, sensitivity increased substantially from 18% (Table 3) to 33%, while specificity remained high. AUC calculated for this two-test model applied to screening referred toddlers only was 0.73.

## Discussion

Debate and controversy regarding the replication of findings from the biological sciences and psychology have been common in recent years [51, 52]. In contrast, the Geometric Preference effect in toddlers with autism has now been replicated multiple times in both direct, identical replication [39] and in this conceptual replication with varied social stimuli. Following our original report in 2011 [38], independent laboratories have also reported similar findings, e.g., [53, 54]. The Complex Social GeoPref test has 97% specificity for ASD in our sample, which is especially high given that our sample contains toddlers with a large variety of presentations beyond typical development and ASD, including language delay and global developmental delay. In comparison, genetic biomarkers of ASD are often pleiotropic and therefore also associated with a number of other neurodevelopmental disorders, so they can have poor specificity [1], as well as low sensitivity due to the large number of different genetic inputs that converge on the ASD phenotype [55]. Effective usage of GeoPref tests would involve prescreening such as we have done here using the CSBS at pediatrician offices; therefore, positive predictive value need not be measured against the 1/68 base rate of ASD in the general population [9]. Without high specificity tests, applied correctly, with results communicated appropriately, false positives do result in inadvertent harms in the process of early identification for infants at risk for ASD [56]. These harms include the family’s exposure to stress and stigma and the unnecessary usage of somewhat scarce and costly intervention services [57]. It has been shown that pediatricians do not refer a significant portion of children who fail screenings for developmental delays, probably due in part to concern regarding the potential for false positive results [58]. Therefore, the availability of screening tools with few false positives could significantly impact the efficacy of screening procedures used for early identification of ASD.

Because the GeoPref tests, both the original and the Complex Social version, detect a subtype of ASD, sensitivity is considerably lower than specificity: at optimal specificity, the Complex Social GeoPref test will catch about 1 in 5 children with autism, while the rate for the original GeoPref test is about 1 in 4.However, here we show that when the two tests are used in combination across separate testing sessions, the correct detection rate is 1 in 3 and specificity remains high at about 94% (Table 4). Further, as can be seen in Fig. 2, the range of %Geo scores for ASD children does not extend as low as that of other groups, which is not a property of the original GeoPref test. If borne out by further data, this could be of value clinically as a means of ruling out ASD in certain children who are exhibiting some ambiguous warning signs but have very low %Geo scores, in order to shift them away from unnecessary ASD services. This result is described in more detail in the Additional file 1. Future research will work toward creating a battery of multiple eye tracking tests in order to further increase sensitivity to ASD in general and to zero in on optimal procedures for detection of this GeoPref subtype of ASD and to elucidate its biological bases.

The ASD GeoPref subtype toddlers detected with GeoPref tests tend to be the most affected cases, as ADOS symptom severity is correlated with %Geo score. It has been observed that more severe presentations of ASD tend to be less studied [59], despite being arguably more in need of treatment. It is possible that the defects impacting the “social brain,” particularly in the frontal regions that control attention and social interest [60], are more pronounced in this ASD subgroup. Since functional brain imaging began to be utilized to understand the operations of the brains of those on the autism spectrum about 20 years ago, abnormalities in virtually every social brain region examined have been reported [61]. However, in addition to the fact that such studies almost exclusively included only older and/or high functioning individuals, data was almost always presented at the group level. As such, previous studies made it hard to understand if reported “social brain” abnormalities were ubiquitous across all ASD individuals, or were being driven by certain subgroups or individuals with the most severe functional abnormalities. In an effort to parse the heterogeneity of social brain neural functional responding in ASD, our new resting state functional imaging study, which examined ASD GeoPref toddlers as a separate subgroup, found substantially weakened functional connectivity between the default mode network (DMN) which includes key “social brain” regions such as the medial prefrontal cortex [62] and a visual network within the occipito-temporal cortex (OTC) in GeoPref ASD toddlers, but not in other toddlers (Lombardo et al., In Review). This finding is consistent with the previous theory that argues that ASD is a disorder wherein higher order social frontal systems are disconnected from more basic systems [63] and further underscores that the severity of this disconnection may be a driving factor in the social abilities of ASD individuals. Notably, ASD toddlers that did not show the GeoPref profile, i.e., SocPref ASD toddlers, did not show distinctly abnormal functional connectivity between the DMN and OTC (Lombardo et al., In Review). The implication of this work is that ASD SocPref toddlers may have stronger and more typical functional circuitry and, again, promise for a better long-term outcome.

Given the intrinsic heterogeneity in the loose category circumscribed by an ASD diagnosis, focusing on subgroups with phenotypic commonalities may be a key research strategy [64]. Another topic for future research is characterization of the GeoPref and SocPref subtypes in terms of traits that are prevalent but not defining characteristics of ASD, such as gastrointestinal issues, altered sensorimotor processing, or comorbid seizure disorder. If found, differences in rates of comorbid epilepsy, motor impairment, and sleep disturbance, because specific mutations have been associated with each [65], could point to genotypic differences between the phenotypic subgroups identified by GeoPref tests.

Consistent with previously reported findings [43], we hypothesized that our revised social stimuli that presented more than one person or social interactions between multiple people tend to magnify the differences between ASD and TD gaze behavior when compared to simpler social stimuli presenting a single person. We did not find this to be the case, as the Original GeoPref test that paired individual children dancing and dynamic geometric images elicited similar or even slightly larger differences between diagnostic groups than the current Complex Social GeoPref test. Alternately, other variables that differ between the Original and Complex Social stimuli, such as salience of biological motion, temporal dynamics of vignettes unfolding, or the overall length, or perhaps differences in low-level visual properties influencing salience (e.g., color or contrast), may account for this finding [66, 67].

One limitation of the current study is that because of differences between the stimuli that are unrelated to their content as social and geometric (e.g., basic feature salience), “geometric preference” is not the only reasonable explanation for the observed differences in behavior across groups. Although we have referred to ASD children who show the least interest in the social stimuli and the most interest in dynamic geometric images as “Geometric Responders” or the “GeoPref” subtype, we have not yet manipulated the geometric images in a large study. It is conceivable that pronounced lack of interest in, or aversion to, the social stimuli alone is driving the geometric preferences, and one could replace the competing stimuli with another type of nonsocial stimuli with similar results. At least one new study suggests that aversion to gaze is not a driving factor in abnormal visual fixation patterns in ASD [68]. However, atypical amygdala responses when viewing eye gaze and faces and disrupted amygdala functional connectivity have been previously observed in ASD and related to gaze aversion and social anxiety [69–71]. It is also possible that the slow rate of saccades shown by the GeoPref subgroups (see Additional file 1) to geometric stimuli may indicate difficulty with attentional disengagement, which then causes longer percent total fixation duration to geometric images. This explanation may have little to do with social motivation, social reward, or “preference” for one stimulus type over the other. Interestingly, however, research studies examining attentional disengagement in ASD are inconclusive, with reports of both deficits [21] and typical responding [72], likely reflecting the wide range of stimuli and procedures used across studies.

The importance of finding the GeoPref profile in toddlers may go beyond its potential value as a screening or even diagnostic biomarker—it may be most importantly useful as a prognostic biomarker. Although it is currently unknown if the abnormal visual fixation patterns displayed during the Complex Social and original GeoPref tests generalize to the everyday life of ASD toddlers, it is at least theoretically plausible that ASD toddlers who display the GeoPref profile are experiencing socially impoverished visual input from their environment. As experience in the first few years of life crucially shapes the brain’s organization, we hypothesize that a GeoPref profile in a toddler may predict distal functional and cognitive outcomes, and our future work intends to examine whether the GeoPref profile is associated with a worse long-term outcome than that of ASD toddlers who prefer social images. Importantly, experience-dependent mechanisms involved in early social learning may be amenable to intervention, and therefore, GeoPref tests may be useful for early identification of and differential intervention for toddlers who strongly attend to certain non-social stimuli, ignoring social information. Tailoring treatment according to ASD subtypes could potentially result in improved treatment responses and better long-term outcomes.

## Conclusion

Across multiple types of social stimuli and temporal presentations, substantially increased viewing of geometric images during preferential looking tasks that pair dynamic social and geometric images robustly indicates ASD among toddlers. Furthermore, across multiple sorts of stimuli, the subset of ASD toddlers who strongly prefer geometric images have more severe scores on indicators of autism impairment compared to those who strongly prefer social images. In addition to replicating the original GeoPref phenomenon, the Complex Social GeoPref test finding shows potential as a valid behavioral biomarker, as it identifies ASD in toddlers at the individual subject level.

## Additional files

Additional file 1: Supplementary Text. (PDF 459 kb)
