# Supplementary material for: The geometric preference subtype in ASD: identifying a consistent, early-emerging phenomenon through eye tracking
Source: Mol Autism. 2018 Mar 21;9:19. doi: 10.1186/s13229-018-0202-z (PMC5861622; doi:10.1186/s13229-018-0202-z)
Supplement: Supplementary file 2 — Supplementary Text. (PDF 459 kb) [file 13229_2018_202_MOESM1_ESM.pdf]

Additional file 1

Additional sample information

Eye tracking data from the Complex Social GeoPref test were collected from 2013 to 2015. If participants were eye tracked multiple times across longitudinal visits, data included are from the earliest appointment available. If clinical assessments were completed multiple times across longitudinal visits, scores included are from the same day when Complex Social GeoPref data were collected whenever possible. Details of the reasons for subject exclusion and number excluded per diagnosis are summarized in Figure S1 below.

Figure S1 Summary of subject inclusion and exclusion. Note: Excluded refers to children for whom eye tracking data was obtained, but was unusable. 4 additional toddlers (2 DD, 1 ASD, 1 Other) would have participated, bringing the total participants to 274, however researchers were unable to obtain any eye tracking data due to inability to calibrate the eye tracker to the child's eye.

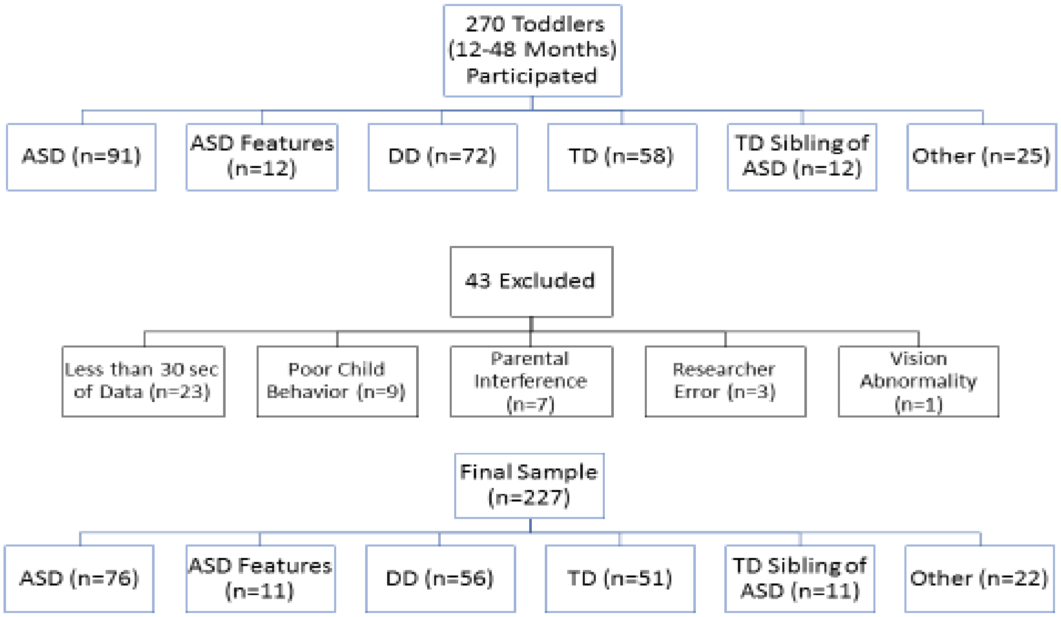

Additional data quality information

Eye tracking data collection typically occurred first or early in an evaluation session, prior to other assessments. Since 2015 we have used spatial accuracy verification testing consisting of a looming cross paired with sound subtending 3.5 degrees at its largest size presented at

three positions (top left, top right, and center) immediately after testing to determine whether excessive loss of spatial accuracy has occurred during the testing process. Though we do not have this information for the current dataset, across 240 toddler testing sessions, we have found that 97% of toddlers test within this margin. For adults we routinely obtain by calibration, and maintain during eye tracking, accuracy with error below 1 degree, and this is verified periodically as part of standard lab practices to assure data quality. Further details of the calibration and eye tracking procedures used were largely the same as those described in our prior paper's supplement [38].

Figure S2 below shows data "heat maps" drawn in Tobii Studio of convenience samples for illustrative purposes. The point of gaze fixation is usually near the AOI's center, so the outer perimeter of the AOI constitutes a large margin of error against spatial accuracy that falls outside the accuracy measurement the initial calibration would predict (e.g. due to drift).

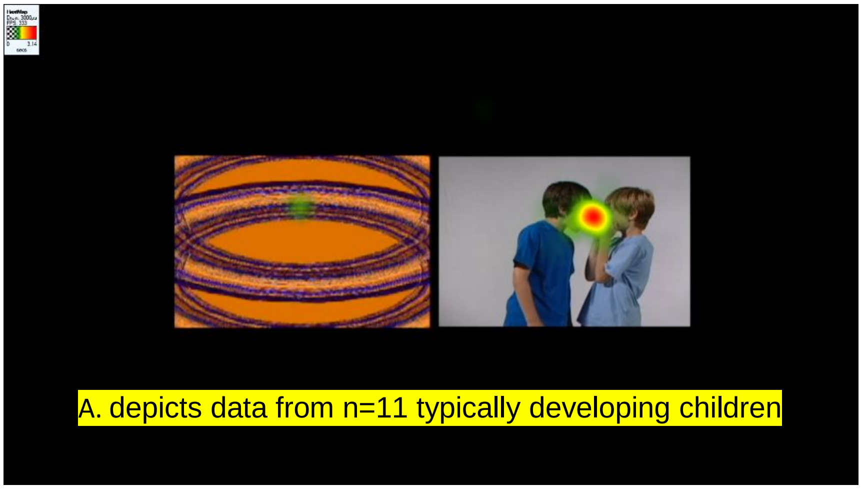

A. depicts data from n=11 typically developing children

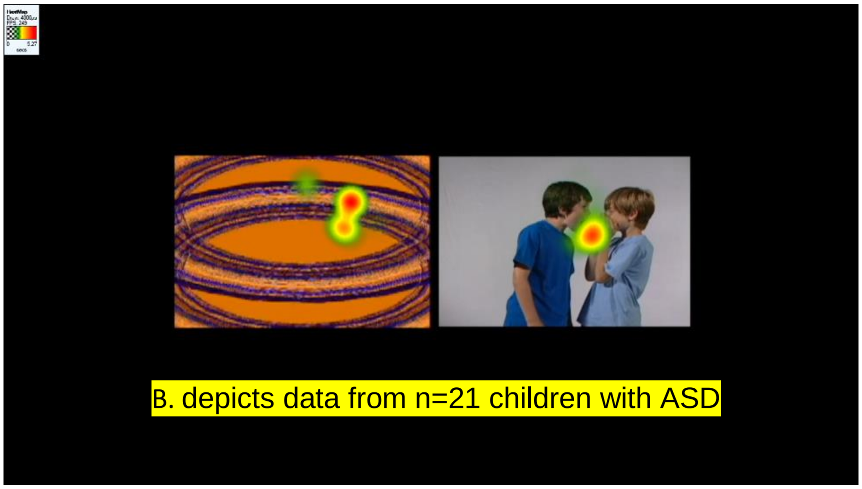

B. depicts data from n=21 children with ASD

The means and standard deviations for total looking time (in seconds, out of a possible total of 90 seconds) to either AOI by diagnostic group were as follows: ASD 65.6 (17.2); ASD Features 79.2 (11.5); DD 76.4 (15.1); TD 78.3 (13.0); Other 77.7 (15.9); TypSib 72.9 (21.6). There was a significant main effect ($F_{5,221}$ =5.8, p<.001) of diagnosis, and significant after Bonferroni correction post-hoc pairwise differences between ASD and the groups DD (p<.005), TD (p<.001) and Other (p<.05). There were no significant differences found between groups in total looking time to the original GeoPref test [38]. The difference found here can likely be attributed to the total duration of the Complex Social test video being 90 seconds, while the original GeoPref test's duration is 60 seconds.

Saccade Frequency to Geometric vs Complex Social Stimuli

Differences in saccades between clinical groups were reported for the original GeoPref test in our 2016 paper [38], therefore we report whether or not these effects were replicated with more complex social stimuli in the current study. For both the geometric and social stimuli, number of fixations per AOI was divided by sum of fixation time for that AOI to derive saccade frequency as saccades per second. Homogeneity of variance was confirmed then 1-way ANOVAs were performed (diagnostic group (6 levels) X saccades/sec (1 level)) for each AOI, and significant effects were followed by pairwise comparisons with Bonferroni correction. To confirm that differences in data quality were not impacting the reported results, ANCOVAs were performed as well, with 6 diagnostic groups as a fixed factor, saccade/sec as the dependent variable, and a data quality measure (percent of valid samples obtained) as a covariate. However, in this case, we found significant effects for the percent samples data quality measure. This implies that variable data quality between groups may be confounding true measurement of saccade rate, so results must be interpreted with caution.

Saccade Frequency of the 6 Groups to Geometric vs Complex Social Stimuli

For each toddler, each stimulus type (geometric and complex social) was considered separately to calculate saccades/sec. Saccade frequency data were subjected to additional scrutiny as this data quality can be impacted by data loss and lack of precision, while overall percent total fixation duration calculations are not as sensitive. Therefore, we excluded five subjects with saccades/sec values greater than two interquartile ranges from the upper quartile, indicating poor fixation filter performance [49]. While looking at geometric stimuli, there were no statistically significant differences in saccades/sec among the six diagnostic groups ($F_{5,216}$=.43,

p=.8). However, ASD toddlers had significantly more saccades/sec when viewing social stimuli than did TD or DD toddlers ($F_{5,216}$=3.4, p=.005, partial eta-squared=.07; ASD vs DD, p<.005, Cohen's d=.63; ASD vs TD, p<.05, Cohen's d=.57). This is consistent with a faster rate of saccades occurring when the child is less interested in or attentive to the stimuli. But this could also reflect less ability to correctly measure saccades from some ASD children (also arguably due to being less interested or attentive).

Saccade Frequency Comparisons within ASD Subgroups

For geometric stimuli, the ASD GeoPref subtype had less frequent saccades, 1.33 saccades/sec, while the ASD SocPref subtype had more frequent saccades, 1.87 saccades/sec, ($t_{25.8}$=2.14, p<.05). For comparison, saccade rates to the same geometric stimuli reported in Pierce et al (2016) for these ASD subgroups were for ASD GeoPref 1.33 saccades/sec, and for ASD SocPref 1.94 saccades/sec. When viewing the new complex social stimuli, the opposite occurred: the ASD GeoPref group had more frequent saccades, 2.38 saccades/sec, and the ASD SocPref group has less frequent saccades, 1.61 saccades/sec, ($t_{18.4}$=-3.77, p=.001). See Figure S3. This is again consistent with faster rate of saccades when less attentive to the stimulus, but also potentially impacted by difficultly precisely measuring saccade frequency during less attentive behavior.

Figure S3 Saccade Frequencies of ASD Subgroups to Complex Social and Geometric Images

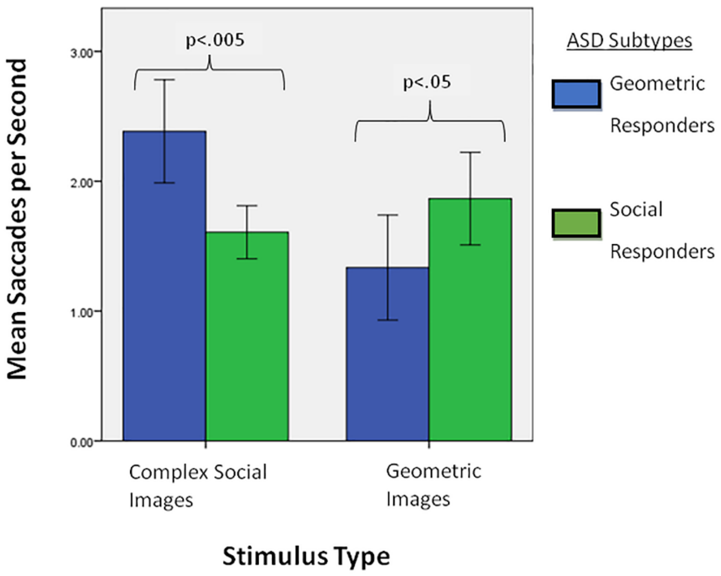

Figure S1. Bar graph illustrating comparison of saccade rate (saccades/second) between two ASD subgroups defined by their % Geo scores in the Complex Social GeoPref test: those who viewed geometric images more than 69% of the time, the Geometric Responder (GeoPref) subtype, and those who viewed social images more than 69% of the time, the Social Responder (SocPref) subtype. Group sizes were n=13 geometric responders and n=16 social responders. Error bars represent 95% confidence intervals.

Additional classification validation statistics

Table S1 shows classification validation statistics based on the cutoff for maximizing specificity with the Complex Social GeoPref test, 75% Geo looking time. At this cutoff, where specificity is 99% (more accurately, it is 99.78%), the PPV of the Complex Social GeoPref test slightly exceeds that of the original GeoPref test, at 92%. That is, on the original GeoPref test, at the 69% cutoff for %Geo the specificity is 99%, however the PPV is 90%. PPV is the likelihood that a given positive test is a true positive, and is therefore of particularly strong interest to clinicians.

Table S1 Clinical Classification Performance, Complex Social GeoPref Test at 99% Specificity

%Geo threshold for positive test = 75%	ASD Only = True Positive<br>N=227
True Positive	12
False Negative	64
False Positive	1
True Negative	150
Sensitivity	16%
Specificity	99%
Positive Predictive Value	92%
Negative Predictive Value	88%
Area Under ROC Curve	.74

Table S2 shows an additional potential usage for the Complex Social GeoPref test, ruling out an ASD diagnosis. That is, children with a %Geo score below 11% (i.e. a %Soc score above 89%) are very unlikely to have an ASD, with test classification performance shown below, where a lower score is more positive. This can be particularly useful as a second tier screen applied to toddlers who have already shown a few potentially concerning behaviors, and who may or may not have an urgent need for an autism focused evaluation. These values were derived by defining positive as falling into any diagnostic group other than ASD, and true positive by also have a %Geo score below 11%; negative refers to having an ASD diagnosis, and true negative to having an ASD and a %Geo score of 11% or greater. In this usage, if a test result is negative, it is inconclusive, because negative results are correct only 36% of the time (NPV=36% below). But if a test result is positive, it is valuable, because 92% of positive results are correct (PPV=92%), and, in this case, 22 toddlers (22 True Positives below) would be correctly identified as not at risk for ASD.

Table S2 Clinical Classification Performance, Complex Social GeoPref Test for Ruling Out ASD

%Geo threshold for negative test = 10%	ASD Only = True Positive N=227
True Positive	22
False Negative	129
False Positive	2
True Negative	74
Sensitivity	15%
Specificity	97%
Positive Predictive Value	92%
Negative Predictive Value	36%
Area Under ROC Curve	.74

Table S3 shows validation classification statistics for detecting children with ASD without the inclusion of typically developing children (i.e. TD or TypSib groups), as distinguishing between ASD children and those with some sort of delay or other challenge that impacts behavior is typically the task facing clinicians. Also, our overall TD group contains some "control" participants, who would not be present in a sample from a non-research clinical setting, so this may be more reflective of a natural clinical sample.

Table S3 Clinical Classification Performance, ASD vs DD and Other

69% Geo threshold	Complex Social GeoPref Test only	Complex Social and Original GeoPref Tests combined	Complex Social and Original GeoPref Tests combined
	ASD Only = True Positive<br><br>vs DD & Other N=154	ASD Only = True Positive<br><br>vs DD & Other N=77	ASD+ ASD Features = True Positive<br>vs DD & Other N=87
True Positive	14	13	15
False Negative	62	24	32
False Positive	2	2	2
True Negative	76	38	38
Sensitivity	18%	35%	32%
Specificity	97%	95%	95%
Positive Predictive Value	88%	87%	88%
Negative Predictive Value	55%	61%	54%
Area Under ROC Curve	.75	.79	.79

Additional clinical severity in relation to eye tracking data information

Figure S3 (below) shows the significant correlation between %Geo scores on the Complex Social GeoPref test and ADOS scores for the entire group of 76 ASD study participants (Pearson’s r=.46, p<.001).

Figure S3

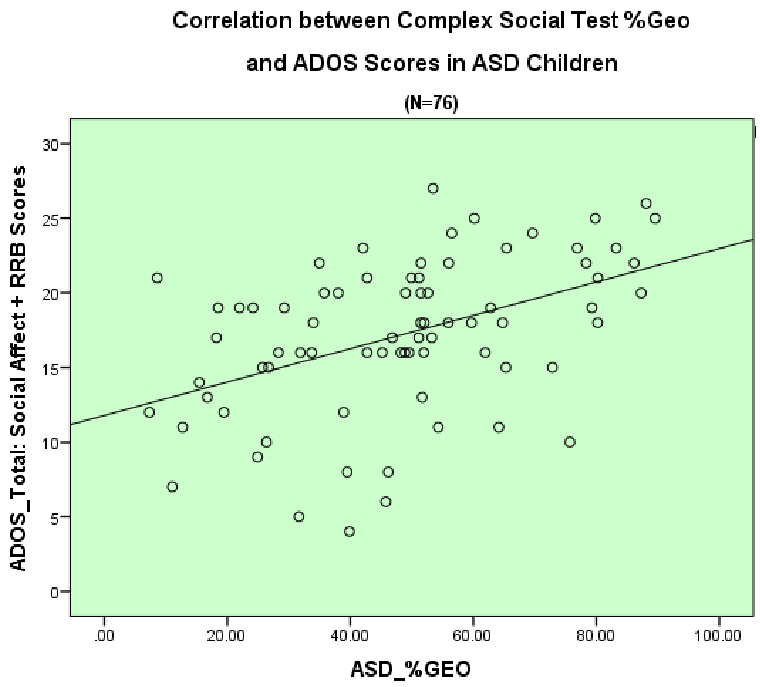

Experiment 2: Exploratory data on back to back usage of the original and Complex Social GeoPref tests

An additional 162 subjects participated in both GeoPref tests administered back to back on the same day. Between tests a break of several seconds was provided and a brief presentation of fixation crosses at known X-Y coordinates was used to confirm continued accuracy of gaze measurement.

Sixty-six of these participants (41%) were excluded from analysis due primarily to behavior incompatible with data collection (excessive movement, tantrums, etc) when exposed

to the combined 2.5 minutes of GeoPref video presentation across two tests. In comparison, only 16% of participants from the main study, when only one GeoPref test was administered on a given day, were excluded. Of the remaining 96 subjects, 41 viewed the Complex Social test first and 55 viewed the original GeoPref test first. All were within the age range of our main study, 12 to 48 months. The diagnoses of the final 96 participants were 28 ASD, 4 ASD features, 32 developmentally delayed, 22 typically developing, 5 typical siblings to ASD, 5 with other diagnoses.

Table S4 below shows classification results for the use of the two GeoPref tests back to back on the same day. While sensitivity, specificity and NPV remain fairly high, PPV drops below 2/3, meaning when a test results is positive there's more than a 1/3 chance that it is incorrect. This value, combined with the large percentage of subjects whose data cannot be used (41%), limits the clinical utility of administering the two GeoPref tests immediately back to back. In the future, the addition of musical sounds to increase willing attentiveness during back to back presentation of two GeoPref videos might be tested.

Table S4 Clinical Classification Performance, Original and Complex Social GeoPref Tests Administered Immediately Back to Back

Positive = positive on either test<br><br>69% Geo threshold	ASD Only = True Positive<br><br>vs all other groups<br>N= 96	ASD+ ASD Features = True Positive<br><br>vs all other groups<br>N=96	ASD Only = True Positive<br><br>vs DD & Other<br>N=65	ASD+ ASD Features = True Positive<br><br>vs DD & Other<br>N=69
True Positive	8	8	8	8
False Negative	20	24	20	24
False Positive	5	5	4	4
True Negative	63	59	33	33
Sensitivity	29%	25%	29%	25%
Specificity	93%	92%	89%	89%
Positive Predictive Value	62%	62%	67%	67%
Negative Predictive Value	76%	71%	62%	58%
Area Under ROC Curve	.76	.74	.74	.71
